# Supplementary figures and images for: HSD17B7 gene in self‐renewal and oncogenicity of keratinocytes from Black versus White populations
Source: EMBO Mol Med. 2021 Jun 29;13(7):e14133. doi: 10.15252/emmm.202114133 (PMC8261506; doi:10.15252/emmm.202114133)

## Source Files - Fig 6D

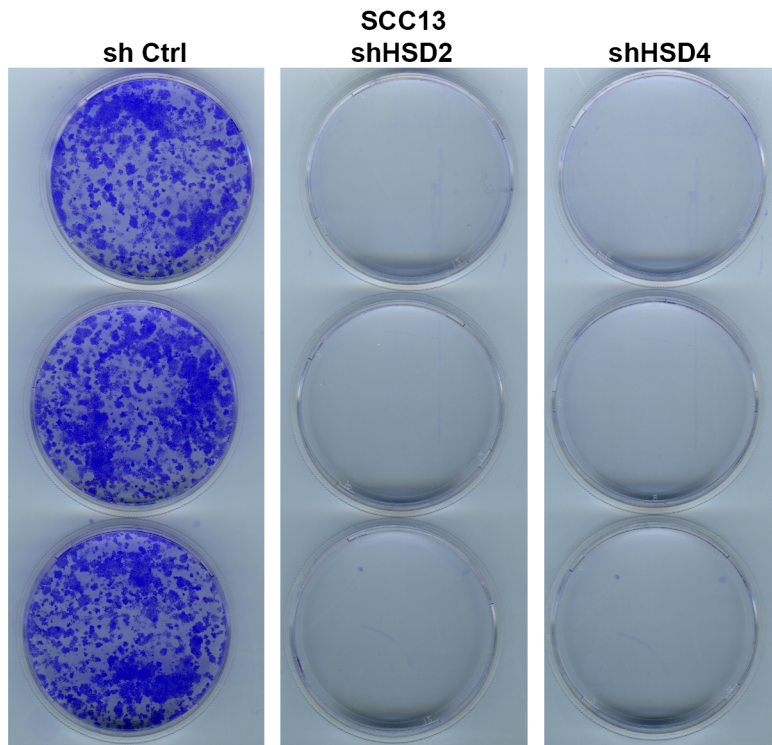

Supplement: Supplementary file 8 — Source Data for Figure 6 [file EMMM-13-e14133-s003.pdf]
