# Supplementary material for: HSD17B7 gene in self‐renewal and oncogenicity of keratinocytes from Black versus White populations
Source: EMBO Mol Med. 2021 Jun 29;13(7):e14133. doi: 10.15252/emmm.202114133 (PMC8261506; doi:10.15252/emmm.202114133)
Supplement: Supplementary file 1 — Appendix [file EMMM-13-e14133-s002.pdf]

**APPENDIX**

***HSD17B7* gene in self-renewal and oncogenicity of keratinocytes from Black versus White populations**

Xiaoying Xu[1+], Beatrice Tassone[1+], Paola Ostano[2], Atul Katarkar[1], Tatiana Proust[1], Jean-Marc Joseph[3], Chiara Riganti[4], Giovanna Chiorino[2], Zoltan Kutalik[5], Karine Lefort[1#] and G. Paolo Dotto[1,6,7]

[*] Correspondence: paolo.dotto@unil.ch

**Table of contents:**

**Appendix Figures and Figure Legends:**

- Appendix Fig S1 Greater oncogenic potential of HKCs from individuals of Back versus White descent (related to Figure 2A-D).
- Appendix Fig S2 Clonogenicity of HKCs from individuals of Back versus White descent and of HKCs and SCC cells plus/minus *HSD17B7* overexpression (related to Fig 2E and Fig 5A,B).
- Appendix Fig S3 Immunofluorescence analysis of HSD17B7 protein expression and localization (related to Fig 4G).
- Appendix Fig S4 Enhanced tumorigenic potential of HKCs with HSD17B7 overexpression (related to Fig 5D-F).
- Appendix Fig S5 HSD17B7 levels in HKCs and SCCs upon silencing or overexpression (related to Fig 6 and Fig 9).

- Appendix Fig S6. Differential mitochondrial OXPHOS activity in Black versus White HKCs (related to Fig 8).
- Appendix Fig S7 Amino acid sequence alignment between HSD17B7 and HSD17B1 catalytic sites (related to Fig 9).
- Appendix Table S1 (related to Fig 2, 4, 5, 6, 8, 9, S6). Individual p values of statistical analyses performed in this study.

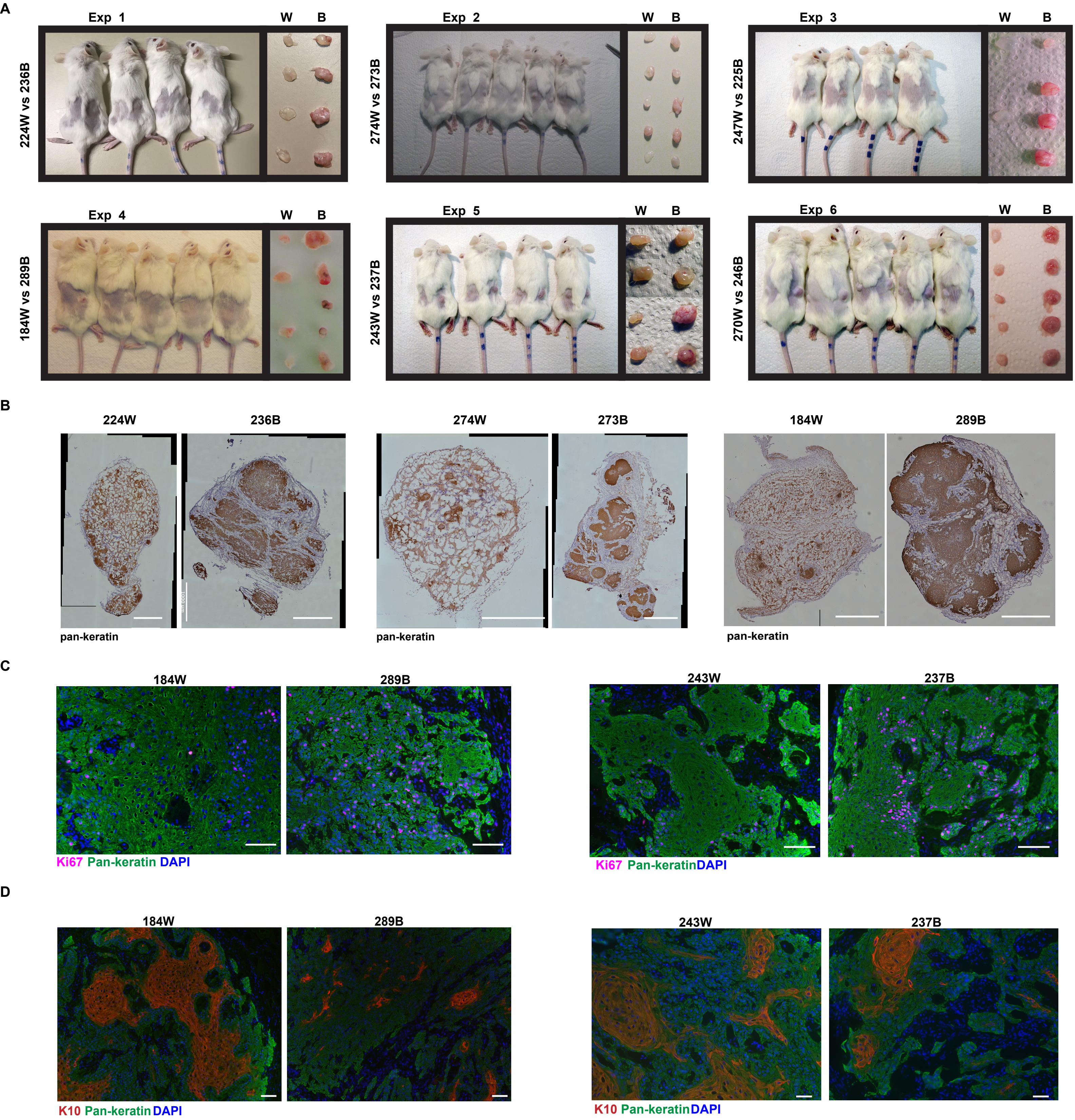


**Appendix Fig S1 Greater oncogenic potential of HKCs from individuals of Back versus White descent (related to Fig 2A-D).**

(A) End result of intradermal tumorigenicity experiments (Exp. 1-6) based, in each case, on parallel mouse injections of HKC strains from Black versus White donors Shown are images of mice together retrieved tumor lesions. Strain numbers and origins (B versus W) are indicated in the figure. HKCs were transduced with TP53R248W and H-RasV12 expressing viruses as described for Fig 2.

(B) Representative images of immunohistochemical analysis of tumors formed by HKC strains of the two ancestries with anti-pan-keratin antibodies in addition to those shown in Fig 2A. Scale bar: 500μm.

(C-D) Representative images of double immunofluorescence analysis of tumors with antibodies against Ki67 (C) and keratin 10 (D) together with anti-pan-keratin antibodies for tumor cells identification, in addition to those shown in Fig 2C, D. Scale bar: 100μm.

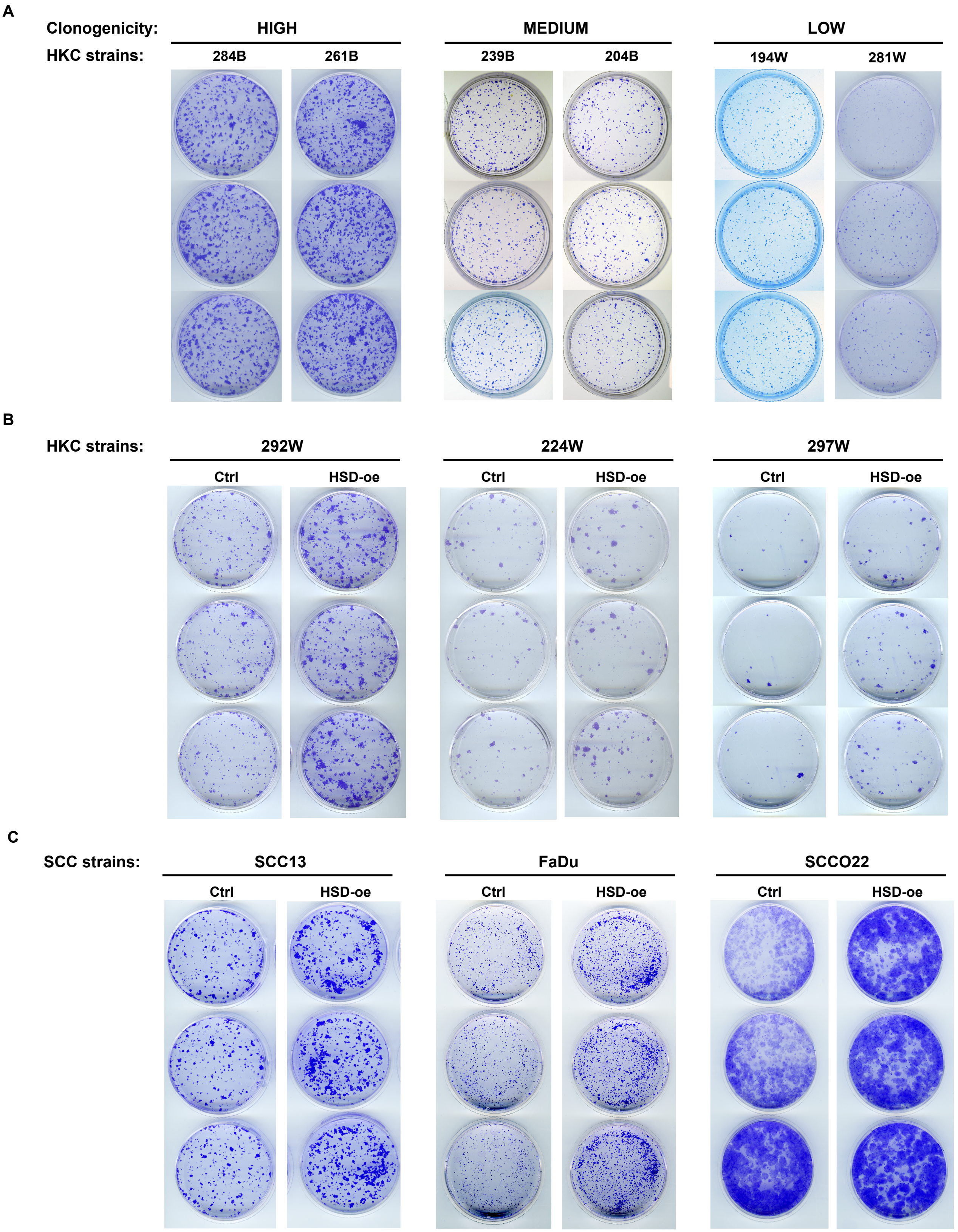


**Appendix Fig S2 Clonogenicity of HKCs from individuals of Back versus White descent and of HKCs and SCC cells plus/minus HSD17B7 overexpression (related to Fig 2E and Fig 5A,B).**

(A) Representative images of clonogenicity assays performed with HKCs with high, medium and low potential as quantified in Fig 2E. either Black or White, divided by clonogenicity (High, Medium, Low). Strain numbers and origins (B versus W) are indicated in the figure.

(B) Representative images of clonogenicity assays with 3 different HKC strains stably infected with a HSD17B7 overexpressing lentivirus (HSD-oe) versus control (Ctrl) as indicated in Fig 5A.

(C) Representative images of clonogenicity assays with three different SCC cell lines stably infected with a HSD17B7 overexpressing lentivirus (HSD-oe) versus control (Ctrl) as indicated in Fig 5B.

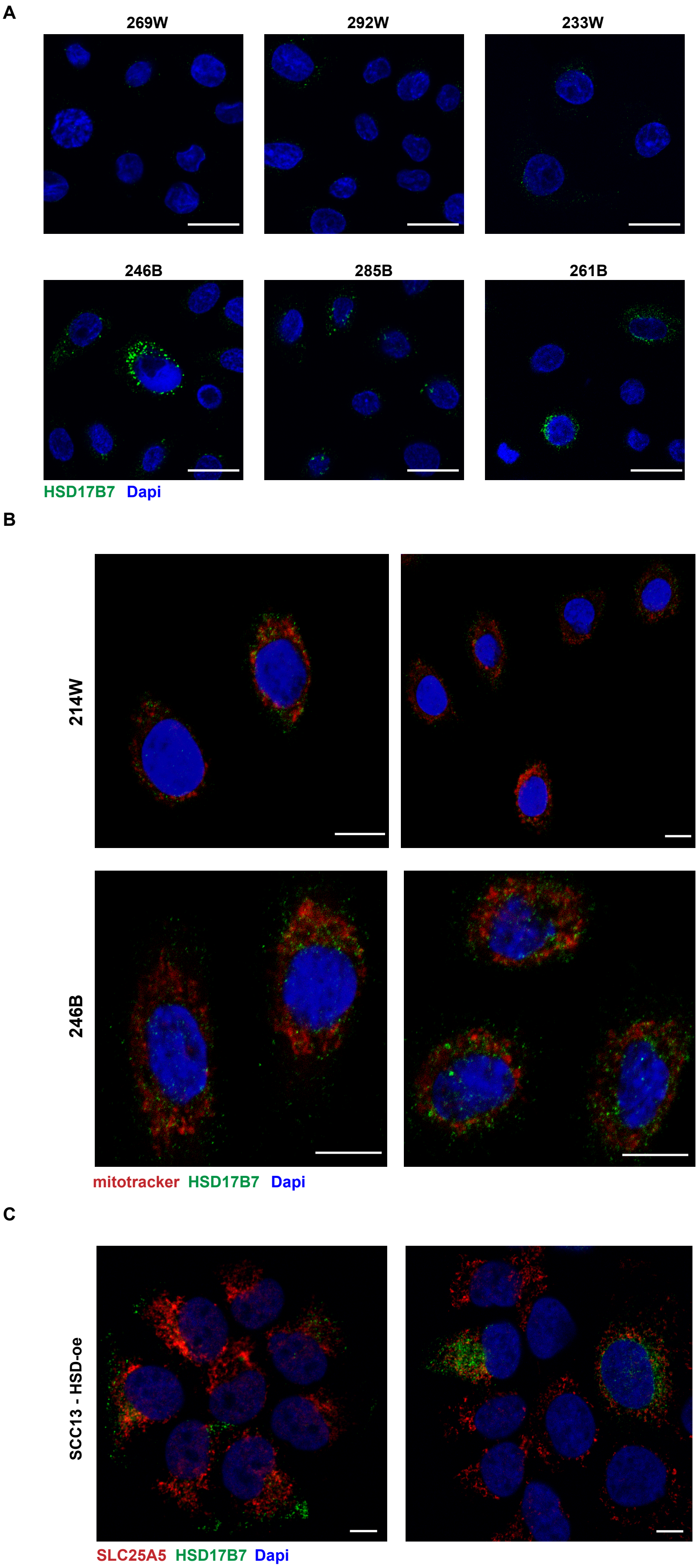


**Appendix Fig S3 Immunofluorescence analysis of HSD17B7 protein expression and localization (related to Fig 4G).**
(A) Immunofluorescence (IF) analysis of HSD17B7 protein expression in White and Black HKCs. Shown are representative images in addition to those shown in Fig 4G. Strain numbers and origins (B versus W) are indicated in the figure. Scale bar: 20µm.
(B) IF analysis of HSD17B7 protein expression in the indicated HKC strains together with MitoTracker staining of mitochondria. Scale bar: 10µm.
(C) Double IF analysis of SCC13 cells stably infected with a HSD17B7 overexpressing lentivirus with antibodies against the HSD17B7 protein and the SLC25A5 mitochondrial marker. Scale bar: 10µm.

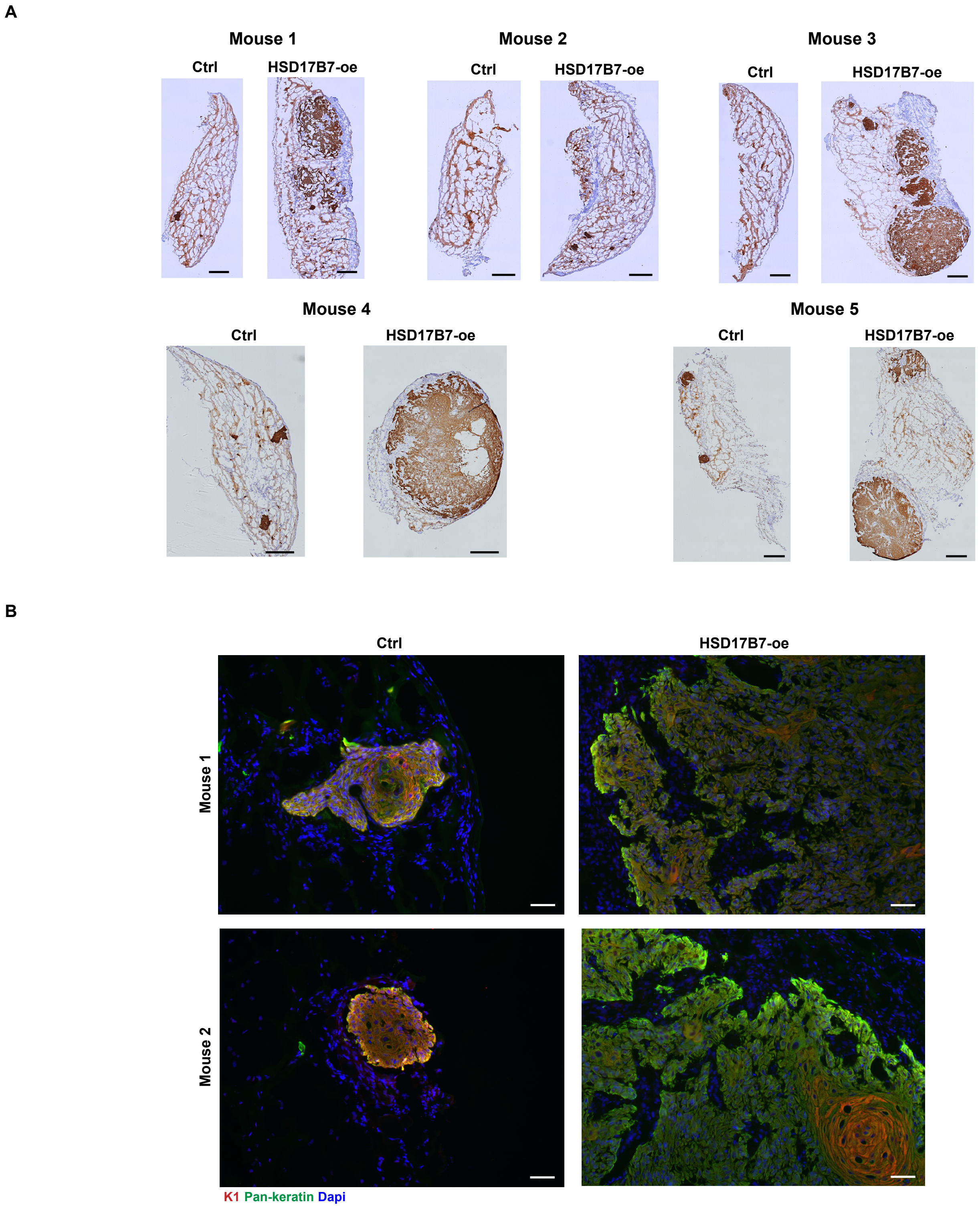


**Appendix Fig S4 Enhanced tumorigenic potential of HKCs with HSD17B7 overexpression (related to Figure 5D-F).**

(A) Immunohistochemical analysis with anti-pan-keratin antibodies of tumors formed by HKCs (269W) stably infected with a HSD17B7 overexpressing (HSD17B7-oe) versus control (Ctrl) lentiviruses and subsequently transduced with TP53R248W and H-RasV12 expressing viruses as in Fig 2A. Shown are images of tumors besides those shown in Fig 5D. Scale bar: 500μm.

(B) Double immunofluorescence analysis of tumors formed by HKCs plus/minus HSD17B7 overexpression (HSD17B7-oe) with antibodies against K1 together with anti-pan-keratin antibody for tumor cells identification. Shown are images of tumors besides those shown in Fig 5F. Scale bar: 50μm.

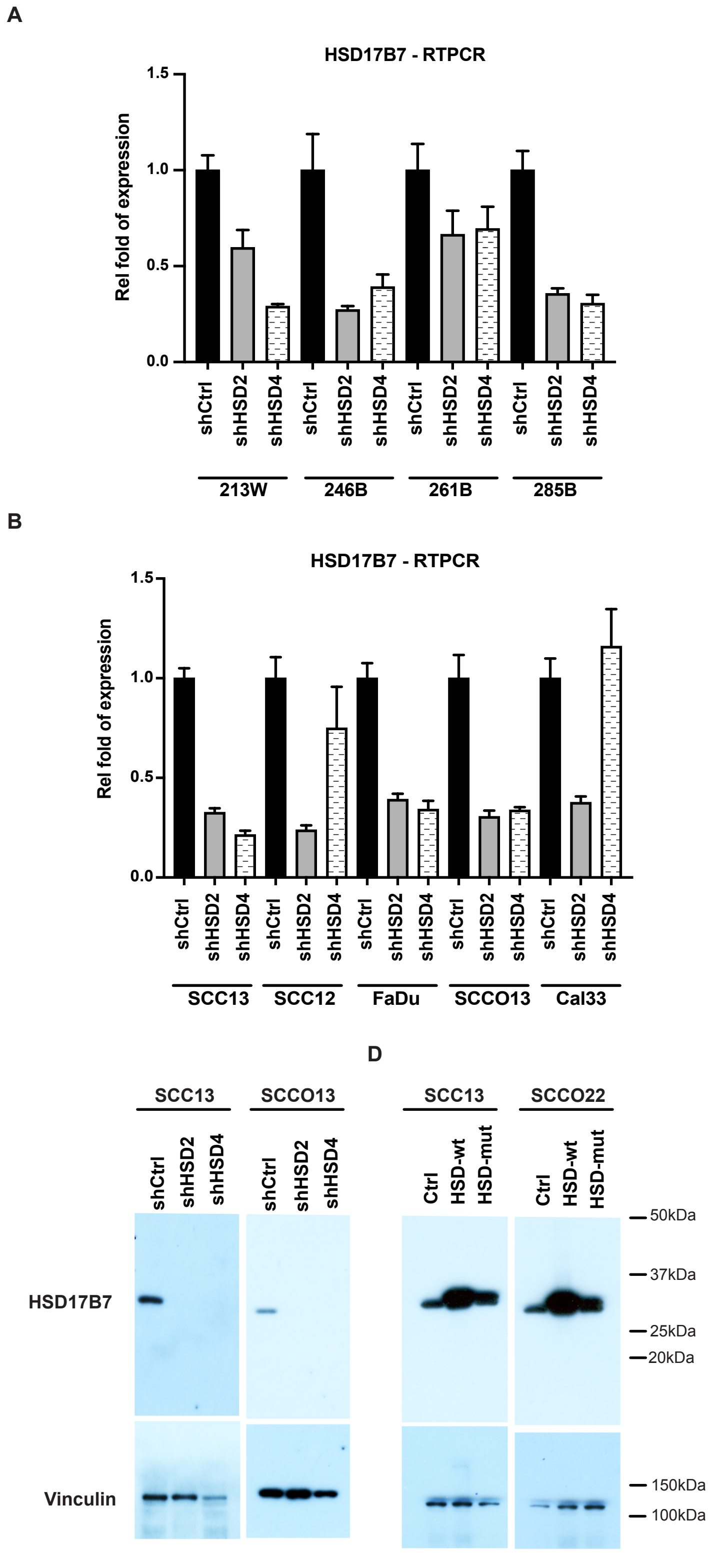


**Appendix Fig S5 HSD17B7 levels in HKCs and SCCs upon silencing or overexpression (related to Fig 6 and Fig 9).**

(A, B) RT-qPCR analysis of HSD17B7 expression in the indicated HKC strains (A) and SCC cell lines (B) at 5 days after infection with two different HSD17B7 silencing lentiviruses (shHSD2 and shHSD4) versus empty vector control (shCtrl). 36B4 expression was used for normalization.

(C) Immunoblot analysis of HSD17B7 protein expression in the indicated SCC cell lines plus/minus infection with two HSD17B7 silencing lentiviruses (shHSD2 and shHSD4) versus empty vector control (shCtrl). Immunoblotting with anti-vinculin antibodies was used for equal loading control.

(D) Immunoblot analysis of HSD17B7 protein expression in the indicated SCC cell lines stably infected with lentiviruses overexpressing HSD17B7 in either wild type (HSD-wt) or mutant forms (HSD-mut), as described in the text, versus empty vector control (Ctrl). Immunoblotting with anti-vinculin antibodies was used for equal loading control.

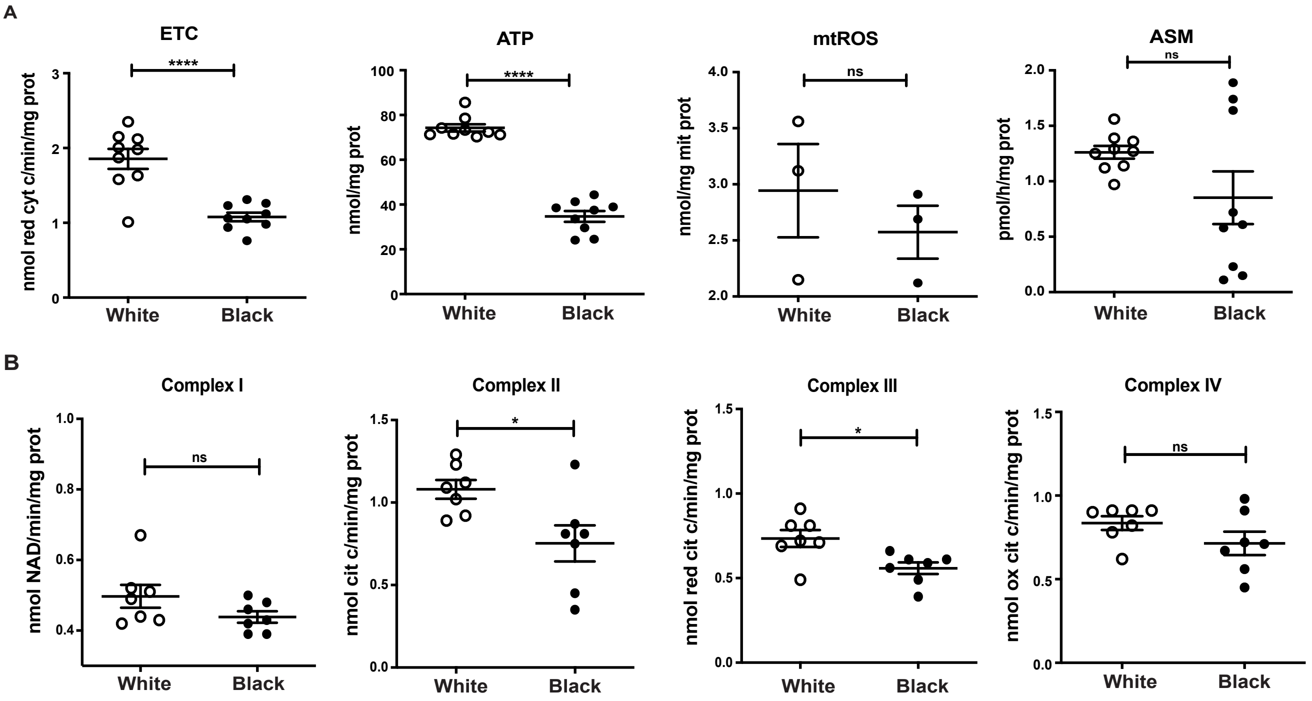


**Appendix Fig S6 Differential mitochondrial OXPHOS activity in Black versus White HKCs (related to Fig 8).**

(A) Purified mitochondrial preparation from multiple HKC strains from White and Black donors (3 per ancestry; passage 4-5; triplicate cultures) were analyzed for levels of Electron Transport Chain activity (ETC, nmol of reduced cytochrome c/min/mg protein), ATP (nmol/mg protein), mitochondrial ROS (MtROS (nmol/mg mitochondrial protein) and Acid Soluble Metabolites (ASM, pmol/h/mg protein). Data are displayed as single values for each culture dish of HKC strains (3 cultures per strain/3 replicates for ETC, ATP and ASM, 1 replicate for mtROS, white and black dots, depending on ancestry) together with mean +/- SD. n(cultures of HKC strains per ancestry)= 9 for ETC, ATP and ASM, 3 for mtROS. ****p<0.00001. 2-tailed unpaired t-test. Individual experimental values are provided in Dataset EV3.

(B) Purified mitochondrial preparation from multiple HKC strains from White and Black donors (7 per ancestry; passage 2,3) were analyzed for activity of single complexes of the Electron Transport Chain, from Complex I to Complex IV. Each dot represent a single HKC strain (1 culture per strain / 7 strains, white and black dots, depending on ancestry) together with mean +/- SD. n(HKC strains per ancestry)=7 *p<0.05. 2-tailed unpaired t-test. Individual experimental values are provided in Dataset EV3.

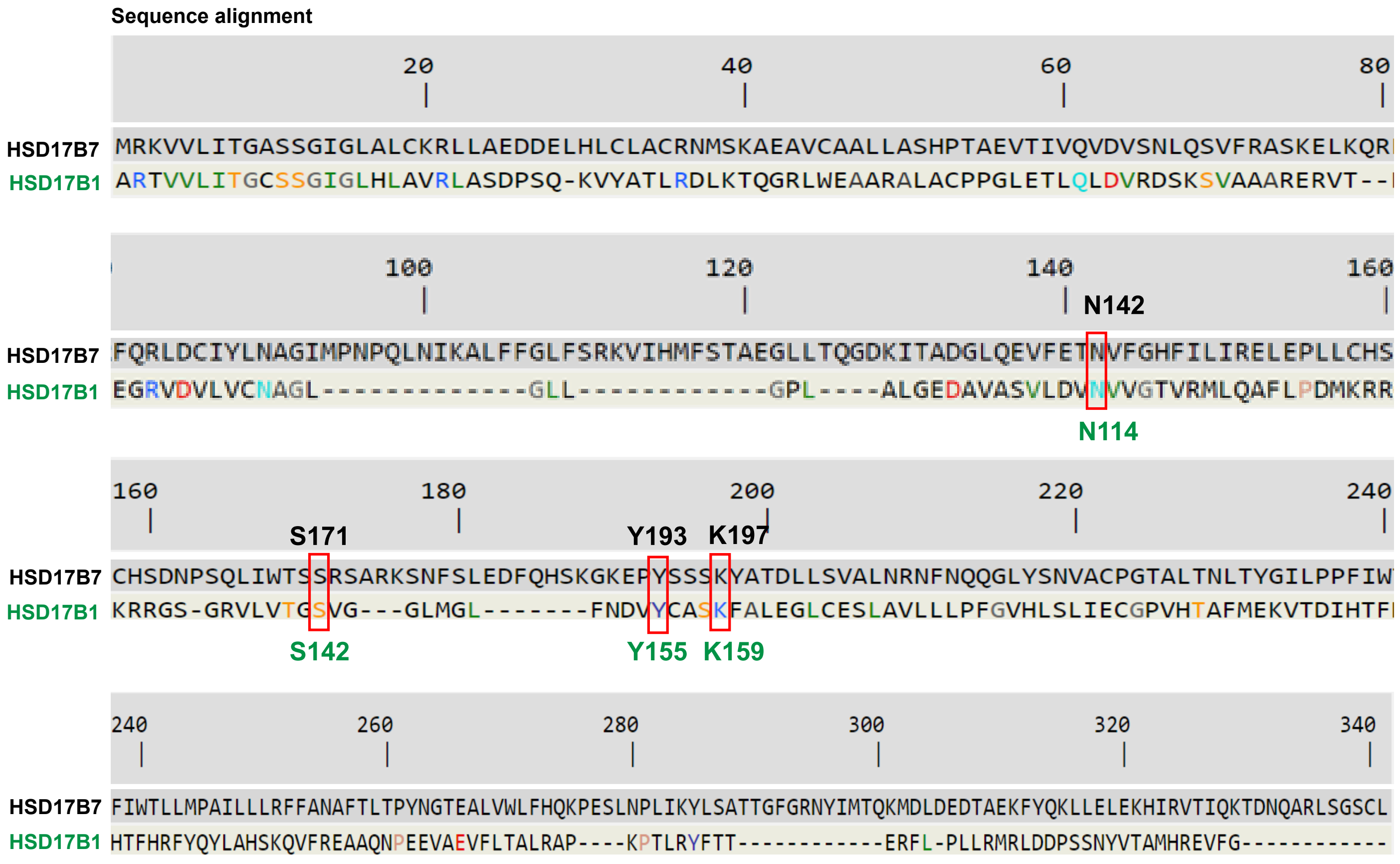


**Appendix Fig S7 Amino acid sequence alignment between HSD17B7 and HSD17B1 catalytic sites (related to Fig 9).** The I-TASSER homology modeling server (https://zhanglab.ccmb.med.umich.edu/I-TASSER/) was used for 3D modeling of the HSD17B7 catalytic site based on the published coordinates of 17-Beta Dehydrogenase 1 (HSD17B1) as template obtained from the Protein Data Bank (https://www.rcsb.org; PDB_ID: 1FDT) (Breton et al., 1996). The sequence alignment and key residues in the catalytic sites (conserved between HSD17B1 (Ser142, Tyr155 and Lys159, in Green) and HSD17B7 (Ser171, Tyr193 and Lys197, in Black) are indicated with red boxes. PyMOL 2.3 software by Schrodinger (https://pymol.org/2/) was used to model visualization and generate images.

**Appendix Table S1**

**(related to Fig 2, 4, 5, 6, 8, 9, S6). Individual pvalues of statistical analyses performed in this study**

**p value data**

**Figure 2**

| | | |
|---|---|---|
| **A** | Black <20% adm vs White | 0.0012 |
| | Black >20% adm vs White | ns |
| | All Blacks vs White | 0.0062 |
| **B** | White vs Black | 0.0205 |
| **C** | Ki67/ White vs Black | 3.20E-05 |
| **D** | K10/ White vs Black | 0.0047 |
| | K1/ White vs Black | 0.0038 |
| **E** | Black<20% adm vs White | 0.003 |
| | Black>20% adm vs White | 1.50E-06 |
| | All Blacks vs White | 1.60E-06 |
| **F** | Black<20% adm vs White | 0.0412 |
| | Black>20% adm vs White | 0.0323 |
| | All Blacks vs White | 0.0088 |

**Figure 4**

| | | |
|---|---|---|
| **F** | White vs Black | 0.009 |
| **G** | White vs Black | 0.0002 |

**Figure 5**

| | | |
|---|---|---|
| **A** | 292W ctrl vs HSD-oe | 0.0386 |
| | 224W ctrl vs HSD-oe | 0.0335 |
| | 297W ctrl vs HSD-oe | 0.0161 |
| | 249W ctrl vs HSD-oe | 0.0372 |
| | 265W ctrl vs HSD-oe | 0.0347 |
| | 269W ctrl vs HSD-oe | ns |
| | 213W ctrl vs HSD-oe | 0.0314 |
| | 225B ctrl vs HSD-oe | ns |
| | 272B ctrl vs HSD-oe | ns |
| | 285B ctrl vs HSD-oe | ns |
| | 237B ctrl vs HSD-oe | ns |
| **B** | SCC13 ctrl vs HSD-oe | 8.00E-05 |
| | FaDu ctrl vs HSD-oe | 0.0147 |
| | SCCO22 ctrl vs HSD-oe | 0.0384 |
| **C** | SCC13 ctrl vs HSD-oe | 1.00E-15 |
| | FaDu ctrl vs HSD-oe | 1.00E-15 |
| | SCCO22 ctrl vs HSD-oe | 1.00E-15 |
| **D** | panK/ ctrl vs HSD-oe | 0.017 |
| **E** | Ki67/ ctrl vs HSD-oe | 6.40E-05 |
| **F** | K1/ ctrl vs HSD-oe | 9.40E-06 |
| **G** | panK/ ctrl vs HSD-oe | 0.0443 |
| **H** | Ki67/ ctrl vs HSD-oe | 0.034 |

**Figure 6**

| | | |
|---|---|---|
| **A** | 213W ctrl vs shHSD2 | 0.0099 |
| | 213W ctrl vs shHSD4 | 0.0065 |
| | 246B ctrl vs shHSD2 | 0.0068 |
| | 246B ctrl vs shHSD4 | 0.0019 |
| | 261B ctrl vs shHSD2 | 0.008 |
| | 261B ctrl vs shHSD4 | 0.0022 |
| | 285B ctrl vs shHSD2 | 0.0045 |
| | 285B ctrl vs shHSD4 | 0.0052 |
| **B** | 246B ctrl vs shHSD2 | 0.0045 |
| | 246B ctrl vs shHSD4 | 3.50E-05 |
| | 261B ctrl vs shHSD2 | 0.0094 |
| | 261B ctrl vs shHSD4 | 0.0038 |
| | 285B ctrl vs shHSD2 | 0.0006 |
| | 285B ctrl vs shHSD4 | 0.0021 |
| **C** | 246B ctrl vs shHSD2 | 0.0003 |
| | 246B ctrl vs shHSD4 | 0.0322 |
| | 261B ctrl vs shHSD2 | 0.0033 |
| | 261B ctrl vs shHSD4 | 8.60E-05 |
| | 285B ctrl vs shHSD2 | 6.80E-09 |
| | 285B ctrl vs shHSD4 | 0.0003 |
| **D** | SCC13 ctrl vs shHSD2 | 4.90E-05 |
| | SCC13 ctrl vs shHSD4 | 6.10E-05 |
| | SCC12 ctrl vs shHSD2 | 9.50E-03 |
| | SCC12 ctrl vs shHSD4 | 1.29E-02 |
| | FaDu ctrl vs shHSD2 | 2.60E-03 |
| | FaDu ctrl vs shHSD4 | 2.60E-03 |
| | SCCO11 ctrl vs shHSD2 | 7.00E-03 |
| | SCCO11 ctrl vs shHSD4 | 5.80E-03 |
| | SCCO13 ctrl vs shHSD2 | 7.70E-03 |
| | SCCO13 ctrl vs shHSD4 | 5.10E-03 |
| | Cal33 ctrl vs shHSD2 | 6.50E-03 |
| | Cal33 ctrl vs shHSD4 | 1.10E-05 |
| **E** | SCC13 ctrl vs shHSD2 | 1.00E-15 |
| | SCC13 ctrl vs shHSD4 | 5.00E-06 |
| | SCC12 ctrl vs shHSD2 | 1.00E-15 |
| | SCC12 ctrl vs shHSD4 | 1.00E-15 |
| | FaDu ctrl vs shHSD2 | 1.00E-15 |
| | FaDu ctrl vs shHSD4 | 6.70E-10 |
| **F** | ctrl vs shHSD2/- | 2.70E-02 |
| | ctrl vs shHSD4/- | 6.70E-03 |
| | shHSD2/- vs shHSD2+HSI | 2.31E-02 |
| | shHSD4/- vs shHSD4+HSI | 2.00E-04 |

**Figure 8**

| | | |
|---|---|---|
| **A** | ETC/ White vs Black | 3.60E-05 |
| | ATP/ White vs Black | 3.10E-06 |
| | MtROS/ White vs Black | 0.0002 |
| | ASM/ White vs Black | 3.70E-05 |
| **B** | ETC/ Ctrl vs HSD-oe | 5.80E-03 |
| | ATP/ Ctrl vs HSD-oe | 0.0109 |
| | MtROS/ Ctrl vs HSD-oe | 9.40E-06 |
| | ASM/ Ctrl vs HSD-oe | 8.60E-08 |
| **C** | ETC/ Ctrl vs HSD-oe | ns |
| | ATP/ Ctrl vs HSD-oe | 0.0028 |
| | MtROS/ Ctrl vs HSD-oe | 0.002 |
| | ASM/ Ctrl vs HSD-oe | 0.0017 |
| **D** | ETC/ shCtrl vs shHSD2 | 1.10E-04 |
| | ETC/ shCtrl vs shHSD4 | 5.40E-05 |
| | ATP/ shCtrl vs shHSD2 | 0.0067 |
| | ATP/ shCtrl vs shHSD4 | 0.0015 |
| | MtROS/ shCtrl vs shHSD2 | 0.0061 |
| | MtROS/ shCtrl vs shHSD4 | 0.0032 |
| | ASM/ shCtrl vs shHSD2 | 0.0046 |
| | ASM/ shCtrl vs shHSD4 | 0.0021 |

**Figure 9**

| | | |
|---|---|---|
| **B** | SCC13*/Ctrl vs HSD-oe | 0.0085 |
| | SCC13*/HSD-oe vs HSD-mut | 0.005 |
| | SCC13/ Ctrl vs HSD-oe | 0.0009 |
| | SCC13/ HSD-oe vs HSD-mut | 0.0052 |
| | SCCO22/Ctrl vs HSD-oe | 0.0132 |
| | SCCO22/HSD-oe vs HSD-mut | 6.30E-05 |
| **C** | ETC/Ctrl vs HSD-oe | 0.0003 |
| | ETC/HSD-oe vs HSD-mut | 0.0005 |
| | ATP/Ctrl vs HSD-oe | 0.0012 |
| | ATP/HSD-oe vs HSD-mut | 0.0008 |
| | MtROS/Ctrl vs HSD-oe | 0.0002 |
| | MtROS/HSD-oe vs HSD-mut | 0.0035 |
| **D** | ETC/vehicle-shHSD2-4 vs zymosterol-shHSD2-4 | 1.70E-07 |
| | ATP/vehicle-shHSD2-4 vs zymosterol-shHSD2-4 | 6.00E-10 |
| | ASM/vehicle-shHSD2-4 vs zymosterol-shHSD2-4 | 1.20E-08 |

**Appendix Figure S6**

| | | |
|---|---|---|
| **A** | ETC/ White vs Black | 0.000067 |
| | ATP/White vs Black | 3.50E-10 |
| | MtROS/White vs Black | ns |
| | ASM/ White vs Black | ns |
| **B** | Complex I/ White vs Black | ns |
| | Complex II/White vs Black | 0.0208 |
| | Complex III/White vs Black | 0.0134 |
| | Complex IV/White vs Black | ns |
